# Complete basic childhood vaccination and associated factors among children aged 12–23 months in East Africa: a multilevel analysis of recent demographic and health surveys

**DOI:** 10.1186/s12889-020-09965-y

**Published:** 2020-12-01

**Authors:** Getayeneh Antehunegn Tesema, Zemenu Tadesse Tessema, Koku Sisay Tamirat, Achamyeleh Birhanu Teshale

**Affiliations:** Department of Epidemiology and Biostatistics, Institute of Public Health, College of Medicine and Health Sciences, University of Gondar, Gondar, Ethiopia

**Keywords:** Complete basic vaccination, Multilevel analysis, East Africa

## Abstract

**Background:**

Complete childhood vaccination remains poor in Sub-Saharan Africa, despite major improvement in childhood vaccination coverage worldwide. Globally, an estimated 2.5 million children die annually from vaccine-preventable diseases. While studies are being conducted in different East African countries, there is limited evidence of complete basic childhood vaccinations and associated factors in East Africa among children aged 12–23 months. Therefore, this study aimed to investigate complete basic childhood vaccinations and associated factors among children aged 12–23 months in East Africa.

**Methods:**

Based on the Demographic and Health Surveys (DHSs) of 12 East African countries (Burundi, Ethiopia, Comoros, Uganda, Rwanda, Tanzania, Mozambique, Madagascar, Zimbabwe, Kenya, Zambia, and Malawi), secondary data analysis was performed. The study included a total weighted sample of 18,811 children aged 12–23 months. The basic childhood vaccination coverage was presented using a bar graph. Multilevel binary logistic regression analysis was fitted for identifying significantly associated factors because the DHS has a hierarchical nature. The Intra-class Correlation Coefficient (ICC), Median Odds Ratio (MOR), Proportional Change in Variance (PCV), and deviance (−2LLR) were used for checking model fitness, and for model comparison. Variable with *p*-value ≤0.2 in the bi-variable multilevel analysis were considered for the multivariable analysis. In the multivariable multilevel analysis, the Adjusted Odds Ratio (AOR) with 95% Confidence Interval (CI) were reported to declare the significance and strength of association with full vaccination.

**Results:**

Complete basic childhood vaccination in East Africa was 69.21% (95% CI, 69.20, 69.21%). In the multivariable multilevel analysis; Mothers aged 25–34 years (AOR = 1.21, 95% CI: 1.10, 1.32), mothers aged 35 years and above (AOR = 1.50, 95% CI: 1.31, 1.71), maternal primary education (AOR = 1.26, 95% CI: 1.15, 1.38), maternal secondary education and above (AOR = 1.54, 95% CI: 1.36, 1.75), husband primary education (AOR = 1.25, 95% CI: 1.13, 1.39), husband secondary education and above (AOR = 1.24, 95% CI: 1.11, 1.40), media exposure (AOR = 1.23, 95% CI: 1.13, 1.33), birth interval of 24–48 months (AOR = 1.28, 95% CI: 1.15, 1.42), birth interval greater than 48 months (AOR = 1.35, 95% CI: 1.21, 1.50), having 1–3 ANC visit (AOR = 3.24, 95% CI: 2.78, 3.77), four and above ANC visit (AOR = 3.68, 95% CI: 3.17, 4.28), PNC visit (AOR = 1.34, 95% CI: 1.23, 1.47), health facility delivery (AOR = 1.48, 95% CI: 1.35, 1.62), large size at birth 1.09 (AOR = 1.09, 95% CI: 1.01, 1.19), being 4–6 births (AOR = 0.83, 95% CI: 0.75, 0.91), being above the sixth birth (AOR = 0.60, 95% CI: 0.52, 0.70), middle wealth index (AOR = 1.16, 95% CI: 1.06, 1.28), rich wealth index (AOR = 1.20, 95% CI: 1.09, 1.33), community poverty (AOR = 1.21, 95% CI: 1.11, 1.32) and country were significantly associated with complete childhood vaccination.

**Conclusions:**

In East Africa, full basic childhood vaccine coverage remains a major public health concern with substantial differences across countries. Complete basic childhood vaccination was significantly associated with maternal age, maternal education, husband education, media exposure, preceding birth interval, number of ANC visits, PNC visits, place of delivery, child-size at birth, parity, wealth index, country, and community poverty. Public health interventions should therefore target children born to uneducated mothers and fathers, poor families, and those who have not used maternal health services to enhance full childhood vaccination to reduce the incidence of child mortality from vaccine-preventable diseases.

## Background

An estimated 5.3 million children under the age of five have died worldwide, the highest in Africa [1]. Children in Sub-Saharan Africa (SSA), particularly East Africa, are more than 15 times more likely than children in high-income countries to die before the age of five [2, 3]. More than half of these deaths are preventable or can be handled with simple, affordable interventions, including immunization, proper nutrition, clean water, and food [4, 5].

Vaccinations against childhood infectious diseases minimize the mortality risk of two-thirds of children under five [6]. Pneumonia and diarrhea, the leading causes of under-five deaths, can be avoided by vaccination [7–9]. Vaccination is the most cost-effective public health strategy for the prevention and eradication of infectious childhood diseases such as measles, pertussis, diphtheria, tetanus, tuberculosis, meningitis, and tuberculosis in children [10–13]. Basic childhood vaccines (BCG, pentavalent, polio, and measles) prevents an estimated 2–3 million deaths of under-five children annually [13–15].

The World Health Organization (WHO) initiated an Extended Program on Immunization (EPI) to establish and extend immunization services worldwide in 1974 to minimize child mortality [16]. Following the launch of the EPI program, the incidence of under-five deaths dropped dramatically from 12.6 million in 1990 to 5.3 million in 2018 [17, 18]. The region with the world’s highest infant and under-five mortality remains SSA, especially East Africa [19], and this could be closely linked with the take-up of vaccinations [20].

In 2018, 13.5 million children were not vaccinated worldwide [21]. Although considerable progress has been made internationally on vaccination coverage, there is a substantial difference in the coverage of vaccines among countries [22]. For European nations, the performance of DPT1 and DPT3 is 97 and 94% respectively, while for Africa, it is 84 and 76% respectively [23]. As for the measles vaccine, it is 95% in developed countries and 74% in Africa [24].

Prior studies on basic childhood vaccination revealed that residence [25–27], maternal occupation status [28, 29], sex of household head [30, 31], wealth status [27, 32], maternal education status [33], husband education status [34], wealth status [27, 35], number of Antenatal Care (ANC) visit [36], place of delivery [27], Postnatal Care (PNC) visit [37], media exposure [35], maternal marital status [27], maternal age [38], parity [39, 40], child-size at birth, mode of delivery [41], twin birth and preceding birth interval were significantly associated factors of complete basic childhood vaccination.

Though there are studies conducted on the prevalence and associated factors of complete basic childhood vaccination in different countries in East Africa [42–45]; as to our search of the literature, there is no study conducted on the complete basic childhood vaccination and associated factors among children aged 12–23 months in East Africa based on the pooled Demographic and Health Surveys (DHSs) data. Investigating complete basic childhood vaccination and its associated factors in East Africa is crucial to assess cross-national disparity in vaccination coverage. Besides, the study had the adequate statistical power to detect the true effects of variables hence the study is based on the pooled DHS data in East Africa. Therefore, this study aimed to investigate the complete basic childhood vaccinations and associated factors among children aged 12–23 months in East Africa using DHS data of 12 countries.

## Methods

### Data source and sampling procedure

This study was based on 12 East African countries’ DHS data. The secondary data analysis was based on the most recent DHS datasets conducted in Burundi, Ethiopia, Comoros, Uganda, Rwanda, Mozambique, Madagascar, Zimbabwe, Kenya, Zambia, Malawi, and Tanzania. These datasets were appended together to investigate complete basic childhood vaccinations and associated factors among children aged 12–23 months in East Africa. The DHSs were a nationally representative survey that collects data on basic health indicators like mortality, morbidity, family planning service utilization, fertility, maternal and child health services (vaccination). The data were derived from the measure DHS program (https://www.dhsprogram.com/Data). Each country’s survey consists of different datasets including men, women, children, birth, and household datasets. For this study, we used the Kids record dataset (KR file). The DHS used two stages of stratified sampling technique to select the study participants. In the first stage, Enumeration Areas (EAs) were randomly selected while in the second stage households were selected. The data were derived from the measure DHS program. We pooled the DHS survey data of the 12 East African countries, and a total weighted sample of 18,811 children aged 12–23 months was included in the study.

### Variables of the study

#### Outcome variable

The dependent variable was the complete basic childhood vaccination status of children aged 12–23 months. As WHO recommended, basic childhood vaccines consists of polio, pentavalent (diphtheria, tetanus, pertussis, haemophiles influenza, and hepatitis B vaccine), measles, and Bacillus Calmette Guerin (BCG) that can prevent common childhood infections. Complete basic childhood vaccination achieved when the child received one dose of BCG vaccine, three doses of pentavalent vaccines, three doses of polio vaccines, and one dose of measles vaccines before the age of 12 months, and categorized as “yes”. While those who failed to take the recommended doses of vaccine were categorized as “no”. The information about child vaccination was derived from the mother’s verbal records and the childhood immunization card data extraction. A random variable Yi represents the i^th^ child’s response variable with two possible values coded as 1 and 0. As a result, the ith child Yi response variable was measured as a dichotomous variable with possible values of “Yi = 1” if the ith child was completely vaccinated and “Yi = 0” if the child was not completely vaccinated.

#### Independent variables

In this study, we considered independent variables at two levels. At level one, the individual-level variables such as maternal age, sex of the child, maternal education, paternal education, media exposure, wealth index, maternal occupation, marital status, sex of head of the household, ANC visit, parity, preceding birth interval, place of delivery, and child-size at birth were included. At level two, community-level variables such as community media exposure, community women education, community poverty, country, and place of residence were considered. In DHS, except residence and country, all the variables are collected at the individual level. Therefore, we generate community-level variables such as community education and community media exposure) by aggregating women’s education and media exposure at the cluster level. It was categorized as low or high using the national median value since these were not normally distributed.

### Data management and analysis

The variables were extracted using the KR file and data cleaning, recoding, and analysis were done using STATA version 14 statistical software. After appending the extracted data from the 12 East African countries, the data were weighted using sampling weight (v005), primary sampling unit (v023), and strata (v021) to draw valid inferences. The proportion of basic childhood vaccination coverage was presented using a bar graph. The DHS data had a hierarchical structure, and this violates the independence of observations and equal variance assumption of the traditional logistic regression model. Hence children and women were nested within a cluster, they may share similar characteristics within the cluster. This implies that there is a need to consider the between cluster variability by using advanced models. Therefore, multilevel binary logistic regression analysis was employed to identify significantly associated factors of complete childhood vaccination. Likelihood Ratio test (LR), Intra-cluster Correlation Coefficient (ICC), Median Odds Ratio (MOR), and Proportional Change in Variance (PCV) were computed to measure the variation between clusters. Model comparison was made based on deviance (−2LLR) since the models were nested. The ICC quantifies the degree of heterogeneity of complete basic childhood vaccination between clusters (the proportion of the total observed difference in complete basic childhood vaccination attributable to cluster variations) [46].

$$ \mathrm{ICC}={\sigma}^2/\left({\sigma}^2+{\pi}^2/3\right). $$

MOR was used to quantify the variation or heterogeneity in complete basic childhood vaccination between clusters. It is defined as the median value of the odds ratio between the cluster high odds of complete basic childhood vaccination and cluster at lower odds of complete basic childhood vaccination when randomly picking out two clusters /EAs [47].

$$ \mathrm{MOR}=\exp .\left(\sqrt{2\ast \partial 2\ast 0.6745}\right)\sim \mathrm{MOR}=\exp .\left(0.95\ast \partial \right). $$

*∂*^2^ indicates that cluster variance.

PCV measures the total variation of complete basic childhood vaccination attributed to individual-level and community-level factors in the final model compared to the null model.

$$ \mathrm{PCV}=\frac{\operatorname{var}.\left(\mathrm{null}\ \mathrm{model}\right)-\operatorname{var}.\mathrm{full}\ \mathrm{model}\left)\right)}{\ \mathrm{Var}\ \left(\mathrm{null}\ \mathrm{model}\right)\ } $$

Four models were constructed for the multilevel logistic regression analysis. The first model (a model without covariates) was the null model, which was done to determine the extent of cluster variation on complete basic childhood vaccinations. The second model (a multilevel model with level-1 independent variables) was adjusted with individual-level variables; the third model (a multilevel model with level-2 variables) was adjusted for community-level variables while the fourth model was fitted with both individual and community level variables simultaneously. Finally, the fourth model was the best-fitted model since it had the lowest deviance value.

Variables with *p*-value ≤0.2 in the bi-variable analysis for both individual and community-level factors were fitted in the multivariable model. Variables with Adjusted Odds Ratio (AOR) with a 95% Confidence Interval (CI), and *p*-value < 0.05 in the multivariable model were reported to declare significantly associated factors of complete basic childhood vaccination. Multi-collinearity was checked using the Variance Inflation Factor (VIF) by doing pseudo-linear regression analysis, which indicates that there was no multicollinearity because all variables have VIF < 5 and tolerance greater than 0.1.

### Ethics consideration

Since the study was a secondary data analysis of publicly available survey data from the MEASURE DHS program, ethical approval, and participant consent were not necessary for this particular study. We requested DHS Program, and permission was granted to download and use the data for this study from http://www.dhsprogram.com. There are no names of individuals or household addresses in the data files.

## Result

### Socio-demographic and economic characteristics of the study population

A total of 18,811 children aged 12–23 months were included, of these 9410 (50.1%) were males. The median age of children was 18 (IQR ± 5) months. Of the total, 3673 (19.5%) of the children were from Kenya and 618 (3.3%) were from Comoros. More than three-fourth (76%) of the children were rural residents. The majority (46.3%) of the children were born to mothers aged 25–34 years, and 24.9% were from mothers who attained secondary education or higher. About 8639 (45.9%) of the children were born to mothers from poor households. Two-third (68.3%) of the children were from the community with low media exposure and 41.2% were from the community with high poverty (Table 1).

**Table 1:** Socio-demographic and economic characteristics of the study population

Variable	Frequency	Percentage (%)
**Country**		
Burundi	1323	7.0
Ethiopia	1945	10.3
Kenya	3673	19.5
Comoros	618	3.3
Madagascar	1139	6.1
Malawi	1066	5.7
Mozambique	2280	12.1
Rwanda	762	4.1
Tanzania	2086	11.1
Uganda	916	4.9
Zambia	1846	9.8
Zimbabwe	1156	6.1
**Residence**		
Rural	14,293	76.0
Urban	4518	24.0
**Sex of child**		
Male	9410	50.1
Female	9401	49.9
**Maternal age**		
15–24	6473	34.4
25–34	8716	46.3
≥ 35	3622	19.3
**Maternal education**		
No	4434	23.6
Primary	9687	51.5
Secondary and above	4690	24.9
**Husband education**		
No	3025	16.1
Primary	7437	39.5
Secondary and above	8349	44.4
**Household wealth status**		
Poor	8639	45.9
Middle	3680	19.6
Rich	6492	34.5
**Marital status**		
Single	1046	5.6
Married	16,224	86.2
Widowed/divorced/separated	1541	8.2
**Sex of household head**		
Male	14,492	77.0
Female	4319	23.0
**Media exposure**		
No	6340	33.7
Yes	12,471	66.3
**Community poverty**		
Low	11,064	58.8
High	7747	41.2
**Community women education**		
Low	14,554	77.4
High	4257	22.6
**Community media exposure**		
Low	12,838	68.3
High	5973	31.7

### Child and maternal-related characteristics

Of the total of 18,811 children, 13,792 (73.3%) were born at the health facility, and 1172 (6.2%) were delivered through cesarean section. About 10,592 (56.3%) of the children were born to mothers who had four and above ANC visits, and 5634 (30%) were from mothers who had PNC checkups (Table 2).

**Table 2:** Child and maternal-related characteristics of children aged 12–23 months in 12 East African countries

Variables	Frequency	Percentage (%)
**Place of delivery**		
Home	5019	26.7
Health facility	13,792	73.3
**ANC visit**		
No	1318	7.0
1–3	6901	36.7
≥ 4	10,592	56.3
**PNC visit**		
No	13,177	70.0
Yes	5634	30.0
**Parity**		
1–3	11,147	59.3
4–6	5420	28.8
≥ 7	2244	11.9
**Child size at birth**		
Large	5323	28.3
Average	8634	45.9
Small	4854	25.8
**Mode of delivery**		
Caesarean delivery	1172	6.2
Vaginal delivery	17,639	93.8
**Birth interval (in months)**		
< 24	2303	12.2
24–48	7911	42.1
> 48	8597	45.7

### Coverage of basic childhood vaccination

**Fig. 1:**
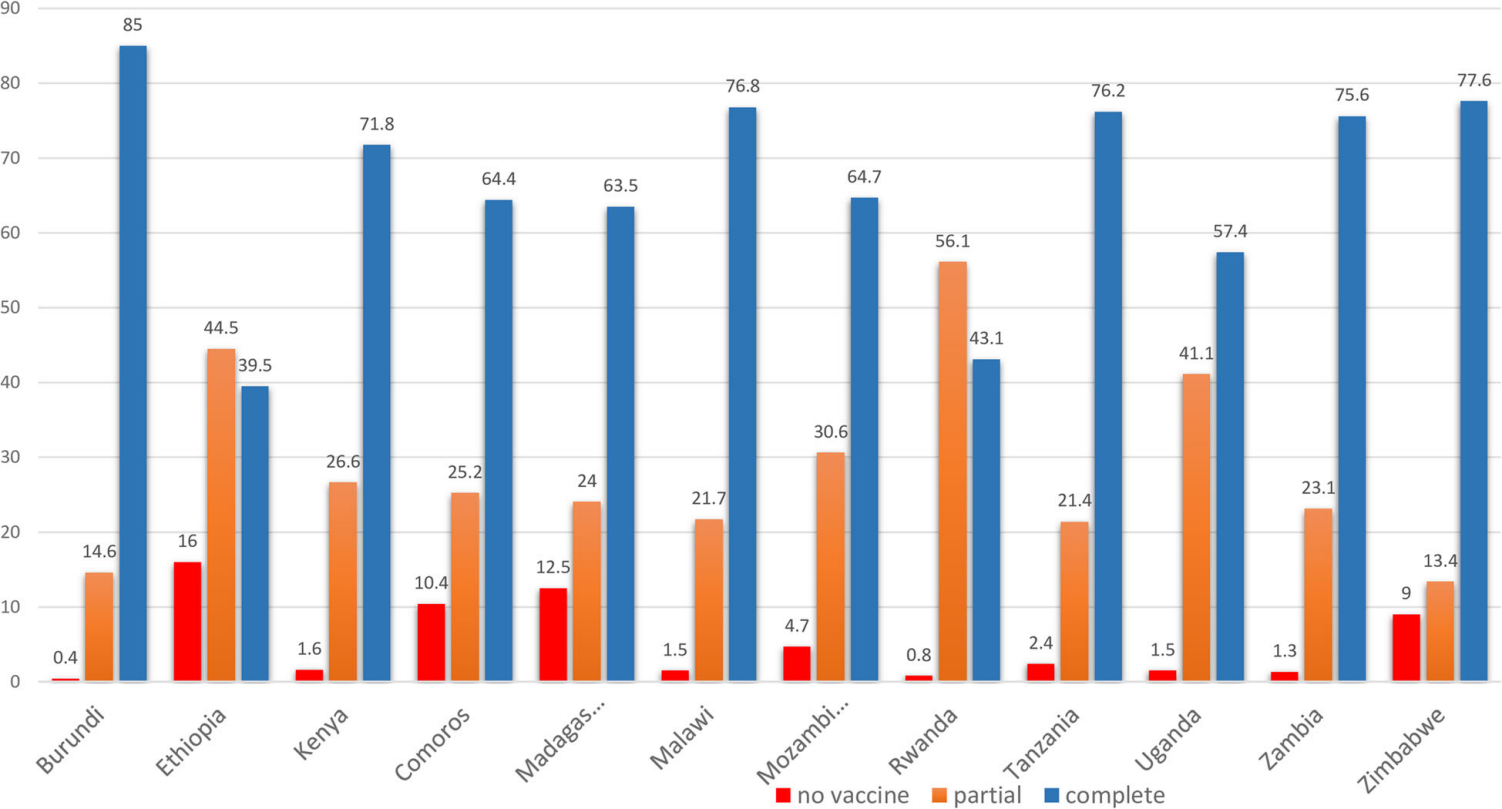
The percentage of basic childhood vaccination status among children aged 12–23 months in East Africa

**Fig. 2:**
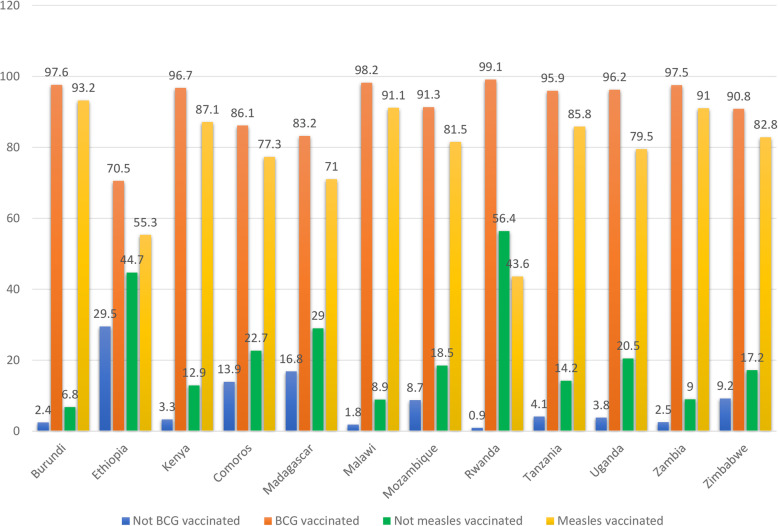
The percentage of BCG and measles vaccination among children aged 12–23 months in East Africa

**Fig. 3:**
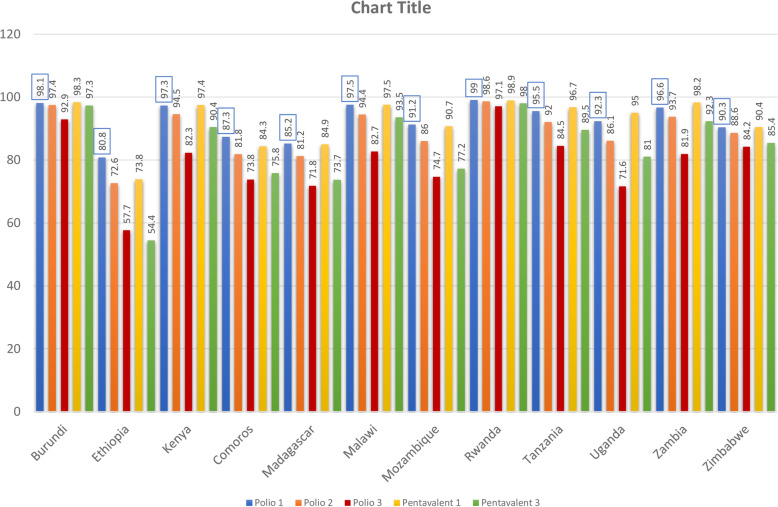
The percentage of polio and pentavalent vaccination among children aged 12–23 months in East Africa

The overall complete basic childhood vaccination among children aged 12–23 months in East Africa was 69.21% (95% CI: 69.20, 69.21%) ranged from 39.5% in Ethiopia to 85% in Burundi. In East Africa, the vaccination status of children has differed greatly across countries. The proportion of partially vaccinated children in Zimbabwe ranged from 13.4 to 56.1% in Rwanda, while the proportion of non-vaccinated children ranged from 0.4% in Burundi to 16% in Ethiopia (Fig. 1). The highest vaccine-specific coverage for BCG was in Rwanda (99.1%), measles was in Burundi (93.2%), polio 3 was in Rwanda (97.1%), and pentavalent 3 was in Rwanda (98%) while the lowest vaccine-specific coverage for BCG was in Ethiopia (70.5%), measles was in Rwanda (43.6%), polio 3 was in Ethiopia (57.7%) and pentavalent 3 was in Ethiopia (54.4%) (Fig. 2 and Fig. 3).

### Associated factors of complete basic childhood vaccination

The ICC in the null model was 0.42 (95% CI: 0.33, 0.55), indicating that the variations between clusters / EAs were responsible for around 42% of the overall variability of complete childhood immunization. Besides, the MOR was 1.45 (95% CI: 1.38, 1.51), meaning that if we randomly pick a child from two separate clusters, a child with a higher probability of complete vaccination in the cluster had a 1.45 times higher probability of being completely vaccinated for the basic vaccines than a child with lower childhood vaccinations in the cluster. as it has the smallest variance value, the final model was the best-fitted model. The PCV in the final model was 0.47, which showed that about 47% of the total variability in the complete basic childhood vaccination was explained by the full model (Table 3).

**Table 3:** Multilevel analysis of individual and community level factors of complete basic childhood vaccination among children aged 12–23 months in East Africa

Variable	Null model (model without independent variables)	Model 1(model adjusted with individual level variables) AOR with 95% CI	Model 2 (model adjusted with community level variables) AOR with 95% CI	Model 3 (model adjusted with individual and community level variables) AOR with 95% CI
**Maternal age**				
15–24		1		1
25–34		1.13 (1.03, 1.23)		1.21 (1.10, 1.32)^*^
≥ 35		1.41 (1.24, 1.61)		1.50 (1.31, 1.71)^*^
**Maternal education**				
No		1		1
Primary		1.22 (1.12, 1.33)		1.26 (1.15, 1.38)^*^
Secondary and above		1.52 (1.35, 1.72)		1.54 (1.36, 1.75)^*^
**Husband education**				
No		1		1
Primary		1.26 (1.14, 1.40)		1.25 (1.13, 1.39)^*^
Secondary and above		1.37 (1.22, 1.53)		1.24 (1.11, 1.40)^*^
**Media exposure**				
No		1		1
Yes		1.15 (1.06, 1.24)		1.23 (1.13, 1.33)^**^
**Preceding birth interval (in months)**				
< 24		1		1
24–48		1.34 (1.21, 1.48)		1.28 (1.15, 1.42)^*^
≥ 49		1.34 (1.20, 1.49)		1.35 (1.21, 1.50)^*^
**Number of ANC visit**				
No visit		1		1
1–3		3.82 (3.30, 4.42)		3.24 (2.78, 3.77)^*^
≥ 4		4.33 (3.74, 5.01)		3.68 (3.17, 4.28)^**^
**PNC visit**				
No		1		1
Yes		1.41 (1.30, 1.52)		1.34 (1.23, 1.47)^*^
**Place of delivery**				
Home		1		1
Health facility		1.52 (1.40, 1.64)		1.48 (1.35, 1.62)^**^
**Mode of delivery**				
Vaginal		1		1
Caesarean		0.97 (0.84, 1.13)		1.05 (0.90, 1.22)
**Child size at birth**				
Large		1		1
Average		1.14 (1.06, 1.24)		1.09 (1.01, 1.19)^**^
Small		1.05 (0.96, 1.15)		0.99 (0.91, 1.10)
**Parity**				
1–3		1		1
4–6		0.88 (0.80, 0.96)		0.83 (0.75, 0.91)^*^
≥ 7		0.64 (0.56, 0.74)		0.60 (0.52, 0.70)^*^
**Marital status**				
Single		1		1
Married		1.19 (1.02, 1.39)		1.14 (0.97, 1.33)
Divorced/widowed/separated		0.88 (0.74, 1.06)		0.85 (0.71, 1.08)
**Wealth status**				
Poor		1		1
Middle		1.22 (1.11, 1.33)		1.16 (1.06, 1.28)^*^
Average		1.21 (1.11, 1.32)		1.20 (1.09, 1.33)^**^
**Country**				
Ethiopia			1	1
Burundi			7.76 (6.48, 9.31)	4.21 (3.47, 5.11)^*^
Kenya			3.04 (2.68, 3.46)	1.96 (1.69, 2.27)^*^
Comoros			2.07 (1.69, 2.52)	1.17 (0.94, 1.45)
Madagascar			2.12 (1.81, 2.48)	1.48 (1.24, 1.76)^*^
Malawi			4.92 (4.13, 5.87)	2.08 (1.72, 2.52)^*^
Mozambique			2.76 (2.41, 3.15)	1.60 (1.37, 1.86)^*^
Rwanda			1.01 (0.84, 1.19)	0.41 (0.34, 0.50)^*^
Tanzania			4.17 (3.62, 4.79)	2.10 (1.79, 2.46)^*^
Uganda			1.85 (1.57, 2.18)	0.91 (0.76, 1.09)
Zambia			4.09 (3.54, 4.73)	1.77 (1.50, 2.10)^*^
Zimbabwe			5.05 (4.22, 6.05)	1.83 (1.49, 2.26)^*^
**Residence**				
Rural			1	1
Urban			1.48 (1.36, 1.60)	0.92 (0.84, 1.02)
**Community women education**				
Low			1	1
High			1.21 (1.09, 1.34)	0.99 (0.89, 1.11)
**Community poverty**				
Low			1.39 (1.27, 1.51)	1.21 (1.11, 1.32)^**^
High			1	1
**Community media exposure**				
Low			1	1
High			1.09 (0.99, 1.20)	0.97 (0.88, 1.07)
**Random effect analysis result**				
Cluster level variance	0.15 (0.11, 0.19)	0.09 (0.06, 0.14)	0.13 (0.09, 0.17)	0.08 (0.06, 0.12)
ICC	0.42 (0.33, 0.55)	0.28 (0.19, 0.39)	0.38 (0.28, 0.51)	0.27 (0.19, 0.40)
LR-test	chibar2(01) = 115.38 p-value< 0.0001	chibar2(01) = 48.85 p-value< 0.0001	chibar2(01) = 83.03 P-value< 0.001	chibar2(01) = 84.51 p-value< 0.0001
MOR	1.45 (1.38, 1.51)	1.33 (1.26, 1.43)	1.40 (1.33, 1.48)	1.31 (1.26, 1.39)
PCV	Ref	0.4	0.13	0.47
LLR	−11,725.223	−10,792.516	−11,029.99	−10,437.633
Deviance	23,450.446	21,585.032	22,059.98	20,875.266

*p-value < 0.05, ** p-value < 0.01, AOR Adjusted Odds Ratio*, CI* Confidence Interval, *ICC* Intra-class Correlation Coefficient, *LLR* Log-likelihood Ratio, *LR* Likelihood Ratio, *MOR* Median Odds Ratio, *PCV* Proportional Change in Variance

In the multivariable multilevel logistic regression analysis; maternal age, maternal education, husband education, media exposure, preceding birth interval, number of ANC visit, PNC visit, place of delivery, child-size at birth, parity wealth status, country, and community poverty were significant associated with complete basic childhood vaccinations.

Mothers aged 25–34 years and ≥ 35 years were 1.21 times (AOR = 1.21, 95% CI: 1.10, 1.32) and 1.50 times (AOR = 1.50, 95% CI: 1.31, 1.71) higher odds of completely vaccinated their children compared to mothers aged 15–24 years, respectively. Children born to mother who attained primary education, and secondary education and above had 1.26 times (AOR = 1.26, 95% CI: 1.15, 1.38) and 1.54 times (AOR = 1.54, 95% CI: 1.36, 1.75) higher likelihood of completely vaccinated than children whose mother did not have formal education, respectively. The odds of being completely vaccinated among children whose father attained primary education, and secondary and above were 1.25 times (AOR = 1.25, 95% CI: 1.13, 1.39) and 1.24 times (AOR = 1.24, 95% CI: 1.11, 1.40) higher than children whose father had no education, respectively. Mothers who had media exposure were 1.23 times (AOR = 1.23, 95% CI: 1.13, 1.33) higher odds of completely vaccinating their children than children born to mothers who didn’t have media exposure. Children born at preceding birth interval of 24–48 months, and greater than 48 months were 1.28 times (AOR = 1.28, 95% CI: 1.15, 1.42) and 1.35 times (AOR = 1.35, 95% CI: 1.21, 1.50) times higher odds of being completely vaccinated than children born less than 24 months of preceding birth, respectively. A child born to mothers who had 1–3 ANC visit, and four and above ANC visit was 3.24 times (AOR = 3.24, 95% CI: 2.78, 3.77) and 3.68 times (AOR = 3.68, 95% CI: 3.17, 4.28) more likelihood of taking complete vaccination than a child born to mother who did not have ANC visit, respectively.

A mother who had a PNC visit was 1.34 times (AOR = 1.34, 95% CI: 1.23, 1.47) higher odds of completely vaccinating their child compared to a mother who did not have a PNC visit, and a mother who gave birth at a health facility had 1.48 times (AOR = 1.48, 95% CI: 1.35, 1.62) higher likelihood of completely vaccinating their children than mother who had home delivery. Mother whose child was average size at birth was 1.09 times (AOR = 1.09, 95% CI: 1.01, 1.19) increased odds of completely vaccinating their children than whose child was large size at birth. The odds of complete vaccination among children born to mother who had 4–6 birth, and above six births were decreased by 17% (AOR = 0.83, 95% CI: 0.75, 0.91) and 40% (AOR = 0.60, 95% CI: 0.52, 0.70) compared to children born to mother who had 1–3 births, respectively. Regarding wealth status, children from the middle and rich households were 1.16 times (AOR = 1.16, 95% CI: 1.06, 1.28) and 1.20 times (AOR = 1.20, 95% CI: 1.09, 1.33) higher odds of complete vaccination compared to children in the poor household wealth respectively.

Among community-level factors; children in Burundi, Kenya, Madagascar, Malawi, Mozambique, Rwanda, Tanzania, Zambia, and Zimbabwe were 4.21 (AOR = 4.21, 95% CI: 3.47, 5.11), 1.96 (AOR = 1.96, 95% CI: 1.69, 2.27), 1.48 (AOR = 1.48, 95% CI: 1.24, 1.76), 2.08 (AOR = 2.08, 95% CI: 1.72, 2.52), 1.60 (AOR = 1.60, 95% CI: 1.37, 1.86), 0.41 (AOR = 0.41, 95% CI: 0.34, 0.50), 2.10 (AOR = 2.10, 95% CI: 1.79, 2.46), 1.77 (AOR = 1.77, 95% CI: 1.50, 2.10) and 1.83 (AOR = 1.83, 95% CI: 1.49, 2.26) times higher odds of complete vaccination compared to children in Ethiopia respectively. Children in the community with low poverty had 1.21 times (AOR = 1.21, 95% CI: 1.11, 1.32) higher odds of complete vaccination compared to children in the community with high poverty (Table 3).

## Discussion

Complete basic childhood vaccination status in East Africa according to the WHO vaccination schedule was low. Childhood full basic vaccination coverage among children aged 12–23 months in East Africa was 69.21% (95% CI: 69.20, 69.21%), which significantly varied across countries. That may be because of inequalities in access to immunization programs and the views of populations about the value of childhood immunization [48]. In East African countries, the ongoing conflict and persistent political instability played an important role in hindering vaccination coverage; the literature showed that lack of safety played a major role in reducing vaccination coverage, especially in remote areas [49]. Besides, there was a high proportion of children vaccinated for BCG, polio 1, and pentavalent 1, but a substantial decline in the proportion of children vaccinated for polio 3, pentavalent 3, and measles. This may be related to vaccine hesitancy in developing countries being a common contributing factor to incomplete vaccination [50]. Cultural misconceptions, adverse effects of vaccinations, and associated consequences could emanate from the root causes of vaccine hesitancy [51]. Also, inadequate management of the adverse effects of vaccinations during regular and supplemental immunization activities may be the reason for not vaccinating their children. For example, measles is often related to mild reactions and is manifested by fever, abscess of the injection site, and irritability [52].

In the multilevel analysis; maternal age, maternal education, husband education, media exposure, preceding birth interval, number of ANC visit, PNC visit, place of delivery, child-size at birth, parity, wealth index, country, and community poverty were significantly associated with complete basic childhood vaccination. Mothers aged 25 and above were more likely than mothers aged 19–24 to completely vaccinate their children. It was consistent with a study reported in Nigeria [27], it may be attributed to the corresponding improvement in the usage of maternal health care services such as ANC visit, health facility delivery, and PNC visit as the entry point for childhood vaccination, as the maternal age rises [53, 54]. Besides, they are aware of lethal childhood diseases that can be avoided by basic vaccinations as the age of the mother rises [55, 56].

In this study, maternal education and husband education were significant predictors of complete basic childhood vaccination. Educated mothers and husbands had higher odds of completely vaccinating their children than uneducated women and husbands. It was in line with studies reported in Nigeria [57], India [58], Indonesia [33], and Turkey [59]. The potential reason may be that maternal and husband education is essential to enhancing the use of primary care services such as childhood vaccination services and increased awareness of childhood immunization [60]. Besides, educated mothers had improved health care decision making autonomy to utilize maternal health care services [61].

Media exposure was significantly associated with increased odds of full childhood vaccination, and it was consistent with studies in Bangladesh [35] and the Democratic Republic of Congo [62]. Media exposure is the most potent health promotion strategy to access the community easily to improve healthcare-seeking behavior [63]. Media exposure plays an essential role in disseminating information about childhood vaccination and allowing behavioral change towards childhood vaccination practices [64].

A mother who used maternal health care services (ANC visit, health facility delivery, and PNC visit) had higher odds of fully childhood vaccination than women who did not use it. It was supported by studies reported in Ghana [65], Senegal [66], and Pakistan [67], it might be due to women who had ANC follow up, health facility delivery, and PNC visit might get counseling service about vaccination [68]. Besides, children born at health facilities get BCG and polio 0 vaccines at birth and get information about the basic childhood vaccination services with their corresponding schedules [69]. This might be the possible reason for the higher odds of full basic childhood vaccinations in women who use maternal health care services during pregnancy, delivery, and delivery.

Women who gave birth before 24 months of preceding birth had lower odds of fully vaccinating their child than women gave birth at 24 months and above. This was consistent with studies in rural Bangladesh [35] and Nigeria [70]. Shorter birth spacing has been associated with increased financial, mental, and psychological consequences to the mothers, and therefore mothers might not use childhood vaccination services [71]. Children who were average size at birth had higher odds of fully immunized compared to children who were large size at birth. It was a consistent study in Nigeria [72], it could be due to mothers who gave average size child at birth are more of interested to keep their child healthy and visit child health care services such as immunization programs than women who gave large size child at birth since the delivery are more of complicated this might hinder their childhood vaccination services utilization [45, 73]. Multiparous women had higher odds of fully vaccinated their children than primiparous women. It was consistent with study findings [74, 75], it might be due to parity is a proxy for the women’s accumulated knowledge of maternal healthcare services utilization from their previous experience, which may have a positive influence on the acceptance of full immunization of children [35, 76].

Children from rich household wealth and low community poverty were more likely to be fully immunized than children from poor household wealth and high community poverty. It supported by previous studies [30, 77, 78], though immunization provided by the EPI program is free, and public efforts to access vulnerable mothers and infants are continued, it might be due to an increase in child care practice, better health-seeking behavior, and health care access among wealthier households [79].

This study has strengths and limitations. This study was based on a pooled nationally representative DHS survey in 12 East African countries that were weighted, and multilevel analysis was done to get a reliable estimate and standard error. Besides, this study was based on a large sample size that had adequate power to detect the true effect of the independent variables. As a limitation, since the study used cross-sectional data, we cannot establish a causal relationship between complete basic childhood vaccination and the identified independent variables. Secondly, Information about the basic vaccinations was collected based on mothers’ verbal responses, besides reviewing vaccination cards. This might cause a lot recall bias since full vaccination status includes BCG, 3 pentavalent, 3 polio and measles vaccines that may overestimate/underestimate the findings.

## Conclusion

Complete basic childhood vaccination coverage remains a major public health problem in East Africa with significant variation across countries. Complete basic childhood vaccination was lower than WHO and UNICEF goals, and it needs substantial progress in improving childhood vaccination and narrowing the gap across countries. Maternal age, maternal education, husband education, media exposure, preceding birth interval, number of ANC visits, PNC visits, place of delivery, child-size at birth, parity, wealth index, country, and community poverty were significant predictors of complete basic childhood immunization. Therefore, public health interventions targeting uneducated mothers and fathers, poor households, and who didn’t use maternal health care services to improve complete childhood vaccination to enhance child survival.

## Data Availability

The datasets used during the current study are available at Measure DHS website: http://www.measuredhs.com.

## Abbreviations

ANC: Antenatal Care
DPT: Diphtheria, Pertussis, and Tetanus
EA: Enumeration Area
DHS: Demography and Health Survey
EPI: Expanded Program of Immunization
ICC: Intra-class Correlation Coefficient
MOR: Median Odds Ratio
PCV: Proportional Change in Variance
PNC: Postnatal Care
SSA: Sub-Saharan Africa
WHO: World Health Organization
